# Malignant Transformation of Schneiderian Papilloma Presenting With Progressive Binocular Diplopia and Blepharoptosis

**DOI:** 10.7759/cureus.10514

**Published:** 2020-09-17

**Authors:** Oraianthi Fiste, Anastasia Tsiogka, Eleni Arvanitou, Athanasios Karampeazis, Charalampos Christofyllakis

**Affiliations:** 1 Department of Clinical Therapeutics, School of Medicine, National and Kapodistrian University of Athens, Athens, GRC; 2 Department of Opthalmology, 401 General Military Hospital of Athens, Athens, GRC; 3 Department of Oncology, 401 General Military Hospital of Athens, Athens, GRC; 4 Department of Oncology, 401 Genaral Military Hospital of Athens, Athens, GRC

**Keywords:** schneiderian papilloma, malignant trasformation, diplopia, blepharoptosis, oncology, ophthalmology

## Abstract

Sinonasal tumors arising from Schneiderian papillomas, most frequently associated with squamous cell carcinoma (SCC), are rare and often present with non-specific symptoms, even in an advanced stage. Herein, we report the case of a 61-year-old male who presented with a four-month history of progressive binocular diplopia, blepharoptosis, and amblyopia, and upon the essential diagnostic work-up he was subsequently diagnosed with SCC arising from an SP. Surgical management was not warranted due to the extent of the disease, so induction chemotherapy with cisplatin and 5-fluorouracil (5-FU) was commenced, followed by definitive concurrent chemoradiotherapy (CRT). The patient was still alive at 25 months after his first presentation, receiving supportive care. Our case highlights the importance of early recognition of neuro-ophthalmological disorders related to sinonasal carcinomas, as diagnostic delay may lead to both functional complications and higher morbidity.

## Introduction

Schneiderian papillomas (SPs) are rare benign, sinonasal tumors [1]. Malignant transformation, albeit uncommon (0.04 cases per 100,000 population) [1], most frequently relates with squamous cell carcinoma (SCC) [2]. Occupational hazards including wood and leather dust have been characterized as causative factors of SP-derived SCCs, whereas, smoking and human papilloma virus (HPV) infection have been suggested to contribute to disease risk [3-5]. Considering their rarity and manifold clinical manifestation [1], early diagnosis poses challenges, whilst optimal management remains an unmet need. In this paper, we describe an additional case of SP-derived SCC presenting with ophthalmological symptoms [6] and critically discuss the published literature on the epidemiology, diagnosis, and treatment of this aggressive malignancy.

## Case presentation

A 61-year-old male was referred to the emergency department with progressive binocular diplopia, blepharoptosis, and amblyopia of four-month duration. He was a heavy smoker with a medical history of moderate alcohol consumption, post-traumatic mydriasis of the right eye, hypertension, and recent intranasal papilloma-plucking for which he underwent a polypectomy excision.

The best corrected distance visual acuity was “hand motion” in the right eye and 1/20 in the left eye (Snellen chart). Ocular motility was abnormal. He had complete ophthalmoplegia with exophthalmos and blepharoptosis in the right eye and sixth nerve palsy in the left eye. Visual fields were abnormal to confrontation visual field examination (Donders' test) with bilateral inferior altitudinal visual field defects.

**Figure 1:**
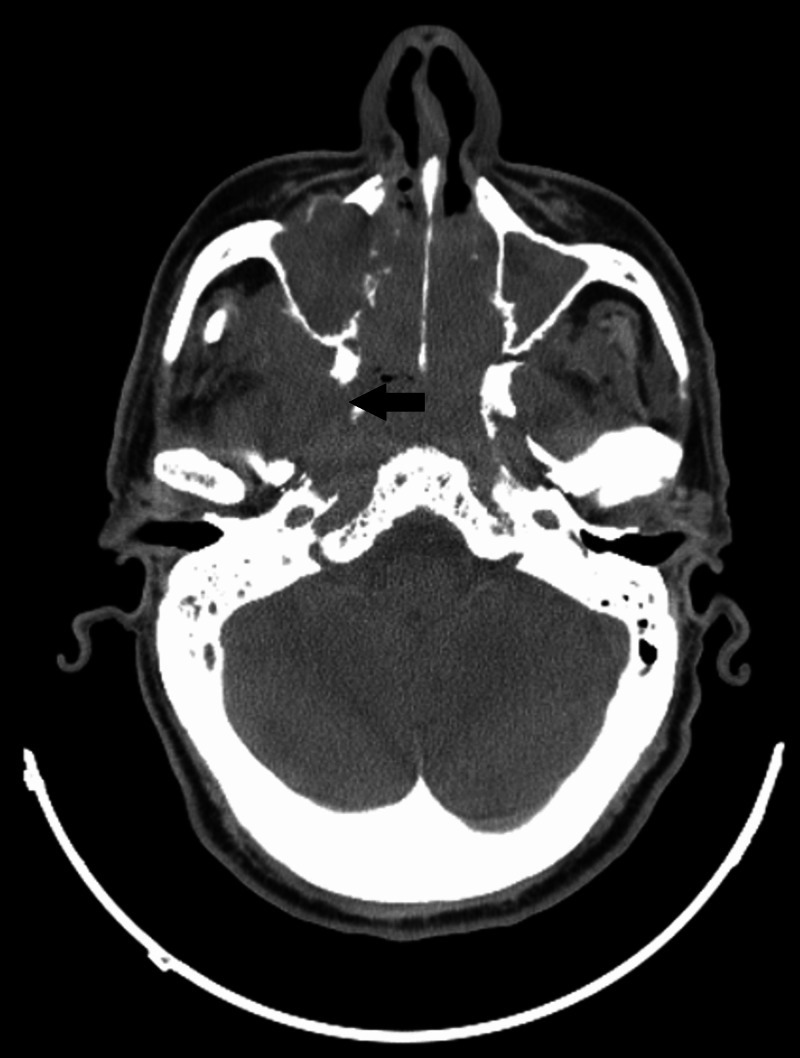
CT scan depicting the tumor (black arrow) from the height of the ethmoidal air cells to the ground of the oropharynx eroding different bones of the area.

**Figure 2:**
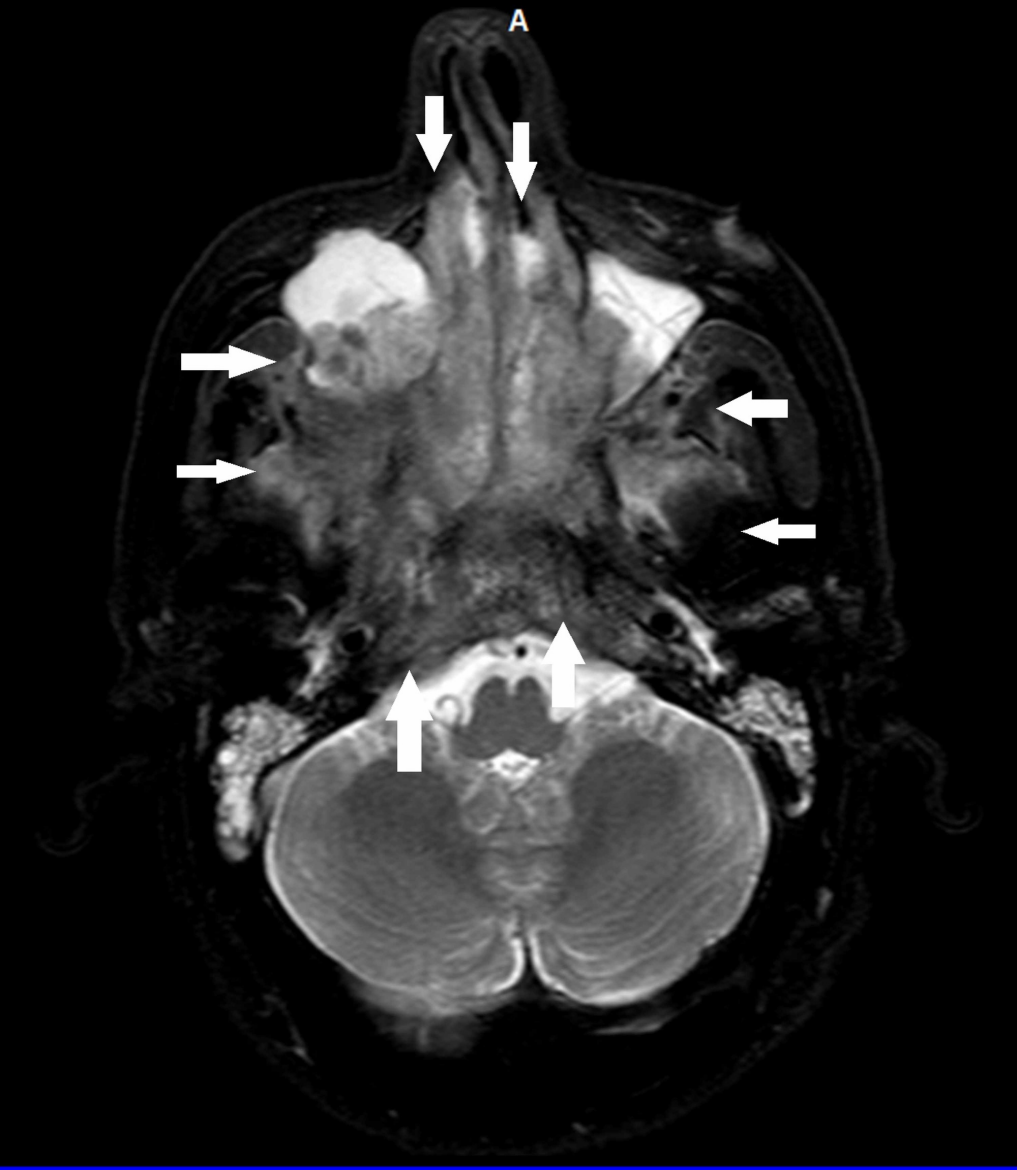
MRI scan depicting the size of the tumor extension (white arrows).

**Figure 3:**
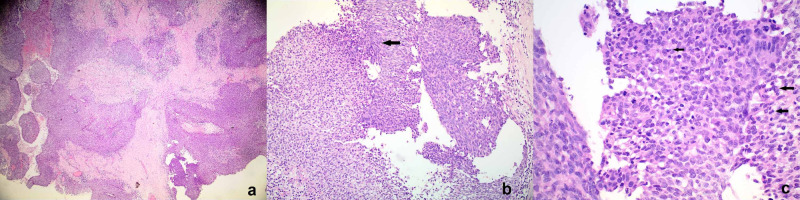
Histopathological examination of the "nasal polyp." (a) Low view of sinosal (Schneiderian) papilloma, inverted and exophytic type, with malignant transformation. (b) Area of transition from the benign epithelium into the area of the malignant epithelium (black arrow). There is increased cellularity, pleomorphism, disorganization, and an increased number of mitoses. (c) High magnification shows the remarkable increased number of mitoses, including atypical forms (black arrows).

Brain imaging techniques, including computed tomography (CT; Figure 1) and magnetic resonance imaging (MRI; Figure 2) both revealed an 11 cm sized mass from the height of the ethmoidal air cells to the ground of the oropharynx, which erodes the maxillary-, the frontal-, the ethmoid-, the sphenoid-bones, and the clivus. Histology on the surgical specimen of the resected nasal polyp, acquired by an ear, nose and throat (ENT) specialist, confirmed the presence of SCC arising from an SP (Figure 3).

Due to the extension of the disease, surgical excision was not feasible; thus, the patient received three cycles of induction chemotherapy with the cisplatin plus 5-fluorouracil (5-FU) combination, followed by radical concurrent chemoradiotherapy (CRT) (63.6 Gy in 30 fractions with weekly cisplatin) with manageable toxicity, partial imaging response, and clinical improvement. The patient was alive 25 months post-diagnosis with progressive disease, receiving the best supportive care.

## Discussion

Schneiderian papillomas (SPs), arising from the ectodermally derived respiratory mucosa, account for 0.5-4.0% of all sinonasal tract tumors [1,5]. They are represented by three morphologically distinct types of papillomas; inverted (ISP), oncocytic (OSP), and exophytic (ESP) sinonasal papilloma, and are classified as benign neoplasms [1]. Malignant transformation of SPs, either synchronous or metachronous, is identified in less than 9% of reported cases, most frequently to SCC [1].

The precise nature of this oncogenic transformation has not been clearly defined; thus, there is an unmet need for further studies into pathogenetic factors of SP-derived SCCs, including the ambiguous role of human papilloma virus type 16 (HPV-16) infection [5,7-9]. Moreover, further evidence is warranted to support tobacco smoking as an etiological factor, despite it relates with increased risk of sinonasal SCCs and is a well-established, modifiable risk factor for head and neck cancer [10]. Also, occupational exposure to wood, leather dust, formaldehyde, nickel, and glues has been strongly associated with tumorigenesis of sinonasal SCCs [3,4,11].

The vast majority of the patients are in general middle-aged males, who present with long symptom duration and advanced-stage disease [1,11,12]. Clinical manifestations of SPs and SP-derived SCCs include unilateral nasal obstruction, epistaxis, headache, sinusitis, anosmia, otitis, vertigo or hearing loss, and ophthalmologic symptoms like diplopia and periorbital swelling [1].

Treatment guidelines for SP-derived SCCs have not yet been established due to their rarity; yet, a multimodality approach including surgery, radiotherapy (RT), and chemotherapy are most commonly employed. Definitive tumor resection, by open craniofacial or endoscopic-assisted approaches, followed by adjuvant RT, results in superior oncologic outcomes compared with CRT, or RT alone, and is considered the optimal treatment of sinonasal SCCs [13-15]. However, in daily clinical practice, the indicated therapeutic modality depends not only on the exact site and extent of the disease but also on the availability of surgical technologies and experience [13]. Nevertheless, the prognosis of SP-derived SCCs remains poor, with an overall five-year survival rate of 30-50% [14]. In the future, the introduction of (a) less invasive and more precise robot-assisted surgery [16], (b) sophisticated RT techniques like intensity-modulated radiation therapy (IMRT), volumetric modulated arc therapy (VMAT), and proton-beam therapy [17-19], and (c) targeted therapies like cetuximab for tumors overexpressing epidermal growth factor receptor [20] might reduce toxicity and increase therapeutic efficiency, thus contribute to significant improvements in both quality of life and survival outcomes.

## Conclusions

SCCs derived from benign SPs are exceedingly rare sinonasal tumors, known to carry a dismal prognosis. Their distinctive characteristics differentiate them from the rest of head and neck cancers, while their clinical presentation includes a broad spectrum of non-specific, atypical symptoms. Diagnosis remains challenging, whereas the complex anatomy of the sinonasal cavity poses therapeutic challenges. Developments in endoscopic surgery, RT, and molecular targeted therapies lay the foundations for optimal management in the near future.
